# DNA Framework‐Based Programmable Atom‐Like Nanoparticles for Non‐Coding RNA Recognition and Differentiation of Cancer Cells

**DOI:** 10.1002/advs.202400492

**Published:** 2024-04-03

**Authors:** Fulin Zhu, Xinyu Yang, Lilin Ouyang, Tiantian Man, Jie Chao, Shengyuan Deng, Dan Zhu, Ying Wan

**Affiliations:** ^1^ School of Mechanical Engineering Nanjing University of Science and Technology 200 Xiaolingwei Street Nanjing 210094 China; ^2^ State Key Laboratory of Organic Electronics and Information Displays & Jiangsu Key Laboratory for Biosensors Institute of Advanced Materials (IAM) Jiangsu National Synergetic Innovation Center for Advanced Materials (SICAM) Nanjing University of Posts and Telecommunications 9 Wenyuan Road Nanjing 210023 China; ^3^ School of Environmental and Biological Engineering Nanjing University of Science and Technology 200 Xiaolingwei Street Nanjing 210094 China

**Keywords:** cancer differentiation, DNA frameworks, fluorescence, programmable atom‐like nanoparticles, RNA recognition

## Abstract

The cooperative diagnosis of non‐coding RNAs (ncRNAs) can accurately reflect the state of cell differentiation and classification, laying the foundation of precision medicine. However, there are still challenges in simultaneous analyses of multiple ncRNAs and the integration of biomarker data for cell typing. In this study, DNA framework‐based programmable atom‐like nanoparticles (PANs) are designed to develop molecular classifiers for intra‐cellular imaging of multiple ncRNAs associated with cell differentiation. The PANs‐based molecular classifier facilitates signal amplification through the catalytic hairpin assembly. The interaction between PAN reporters and ncRNAs enables high‐fidelity conversion of ncRNAs expression level into binding events, and the assessment of in situ ncRNAs levels via measurement of the fluorescent signal changes of PAN reporters. Compared to non‐amplified methods, the detection limits of PANs are reduced by four orders of magnitude. Using human gastric cancer cell lines as a model system, the PANs‐based molecular classifier demonstrates its capacity to measure multiple ncRNAs in living cells and assesses the degree of cell differentiation. This approach can serve as a universal strategy for the classification of cancer cells during malignant transformation and tumor progression.

## Graphical abstract

DNA framework‐based programmable atom‐like nanoparticles (PANs) are used to develop molecular classifiers. These PANs are composed of a DNA octahedral scaffold serving as the core, which integrates multiple functional modules. The PAN reporters are employed to image multiple ncRNAs through catalytic hairpin assembly (CHA) in various cell lines, providing a more precise diagnosis of tumors by distinguishing cancer cell differentiation.

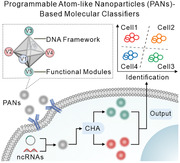

## 1 Introduction

Non‐coding RNAs (ncRNAs) are RNAs that do not code for proteins but regulate important life activity processes in diverse ways.^[^ ^1^ ^]^ With the deepening of ncRNA research, increasing functions of ncRNA are uncovered in gene expression regulation, chromatin remodeling, membraneless organelle assembly, and other important biological processes. Dysregulation of ncRNAs thus leads to various human diseases (e.g., cancer, autism, neurological disorders, and cardiovascular problems), which can serve as promising diagnostic biomarkers and therapeutic targets.^[^ ^2^ ^]^ As multiple ncRNAs work together to regulate cellular activities, the abnormal cell is often accompanied by the abnormal expression of more than one ncRNA. For instance, the differentiation of gastric cancer cells is often accompanied by an upregulation of miRNA‐107 and a downregulation of circHIPK3.^[^ ^3^ ^]^ Evaluating the expression levels of multiple ncRNAs in cells can enable a more precise classification of cell differentiations status, thus leading to an accurate reflection of the clinical behavior of tumors.

Investigating ncRNAs requires the development of precision and efficient analysis tools. Currently, the detection of ncRNAs often relies on techniques such as quantitative real‐time PCR (qRT‐PCR), microarray‐based hybridization, electrochemical measurement, and other new technologies.^[^ ^4^ ^]^ However, these in vitro techniques require the extraction RNA samples from cell lysates and do not allow for real‐time imaging analysis of endogenous ncRNAs.^[^ ^5^ ^]^ As ncRNAs are expressed in a tissue‐ or cell‐type specific manner, it is necessary to develop quantitative methods in living cells. To address this limitation, researchers have explored the use of fluorescent DNA probes to measure ncRNA levels in living cells.^[^ ^6^ ^]^ For instance, nuclease‐resistant molecular beacons have been used to track ncRNAs in live cells. Nevertheless, this technology requires the use of transfection reagents to allow molecule beacons to enter cells. Nanomaterials can be taken up by cells easily, and have been utilized to encapsulate DNA molecules and fluorescent tags to form nanoprobes.^[^ ^7^ ^]^ Despite the successful utility of these nanoprobes in RNA imaging, they introduce foreign substances that may cause cytotoxicity and uncontrollable modification. Moreover, most of the existing methods targeting a single ncRNA may not provide reliable and comprehensive information due to the heterogeneous expression of ncRNAs.^[^ ^8^ ^]^

To simultaneously analyze different types of ncRNA with different lengths and secondary structures in live cells, there are several issues to be addressed: i) The high sequence homology among ncRNAs necessitates the design of a rational recognition module to avoid interference between them. ii) Efficient carriers are needed to deliver multiple functional modules into cells. iii) Given the trace level of ncRNA expression in cells, selective signal enhancement is required to improve the accuracy of detection. Developing multidimensional molecular classifiers can present an efficient means for cell typing based on multiple ncRNAs. Recently, the integration of DNA‐based molecular recognition with molecular classifiers has emerged as a powerful and potentially versatile method for classification.^[^ ^9^ ^]^ Nevertheless, constructing highly integrated molecular classifiers to process multiple molecular data for cell typing remains a challenge.

**Figure 1:**
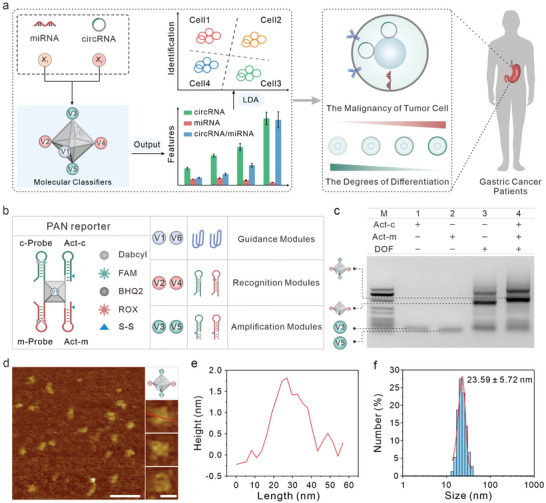
A stimuli‐responsive PAN‐based molecular classifier. a) The DNA octahedral framework (DOF) based‐PANs enabled the construction of molecular classifiers for the analysis of multiple ncRNAs and accurate classification of cancer cells. b) The design of the PAN reporter. c) Agarose gel electrophoresis characterization of PAN reporters. Lane 1–4 represent Act‐c, Act‐m, DOF, and PAN reporters, respectively. d) The structure of PANs with representative AFM images. Scale bar in the left image: 200 nm. Scale bar in the right images: 20 nm. e) The height‐length of the PAN reporter was measured from the red line profiles. f) DLS analysis of PAN reporters.

Here, programmable atom‐like nanoparticles (PANs) are composed of scaffold nanostructure (the “atom”) and functionalized DNAs (the “bonding elements”). Unlike atomic systems, PANs enable independent tuning of the “atom” and the “bond”, facilitating the conversion of multidimensional molecular information into a unified output signal in a programmable manner.^[^ ^10^ ^]^ The precisely defined geometries and nanometer‐scale addressability of DNA framework structures provide a powerful platform for constructing PANs endowed with programmability and directionality.^[^ ^11^ ^]^ Herein, we developed a versatile PANs‐based molecular classifiers employing DNA octahedral framework (DOF) for multiple ncRNA imaging in live cells and further classification of tumor cell differentiation status (**Figure** **1**a). Leveraging the atom‐like and programmable characteristics of DNA framework, we selected DOF scaffolds to serve as atom equivalents in PANs. The ssDNA of DOF scaffold, analogous to the valence bonds of atoms, is the ideal ligand to confer nanoparticles with specific chemical or topological properties. These ligands can direct core nanoparticle bonding with functional modules (functionalized DNA strands) in a manner analogous to atomic bonding, facilitating the construction of PANs‐based molecular classifiers. The PANs‐based molecular classifier strategically incorporates a functional module on each of the six vertices of DOF (Figure 1b). The six functional modules could be divided into three groups: guidance modules (V1, V6), recognition modules (V2, V4) and amplification modules (V3, V5). The guidance modules guide the PAN‐molecules to cancer cells, the recognition modules specifically bind with target ncRNAs and the amplification modules can be specifically activated by glutathione (GSH) within tumor cells. Taking circHIPK3 and miR‐107 as analytes, the as‐fabricated PANs were applied in several gastric cancer (GC) cell lines. By recording and analyzing these fluorescent signals, we obtained characteristic fingerprints that differentiate between cancer cells with varying degrees of differentiation. Due to its excellent classification accuracy, linear discriminant analysis (LDA) was utilized to analyze the obtained data matrices, enabling cell classification while preserving crucial information.^[^ ^12^ ^]^ This approach presents a promising opportunity to accurately classify cancer cell differentiations by profiling multiple ncRNAs simultaneously, which provides a basis for the precision diagnosis and treatment of tumors in clinical practice.

## 2 Results and Discussion

### 2.1 Design and Characterization of the Stimuli‐Responsive PANs

We first designed the PANs with functional modules based on DOFs (Figure 1b). The PANs were constructed in two steps. In the first step, the DOFs were assembled via Watson–Crick base pairing using eight DNA strands to form the atom core building block of the PANs (sequences listed in Table S1, Supporting Information). Each vertex (V1–V6) of the DOFs was assigned a unique overhang. Two overhangs were designed at the opposite vertices (V1, V6) of the DOF using AS1411 aptamer sequences as guidance modules. The AS1411 aptamer was known to bind with nucleolin, which is overexpressed on the surface of cancer cells, thereby facilitating the internalization of PANs into the cancer cells.^[^ ^13^ ^]^ At the vertices (V2, V4), we introduced two stem‐loop structures (c‐Probe and m‐Probe), each labeled with FAM/Dabcyl and ROX/BHQ2, respectively. These stem‐loop structures functioned as recognition modules, enabling the detection of ncRNAs through changes in fluorescence signals. The overhangs at the remaining opposite vertices (V3, V5) were utilized in the next step to hybridize with the toeholds of Act‐c and Act‐m, respectively, resulting in the formation of the PANs reporter. Notably, the Act‐c and Act‐m as amplification modules contain a disulfide bond (S−S) that could respond to GSH stimulation. In the presence of GSH, the amplification modules were disassembled from the PANs via disulfide cleavage by GSH. The dissociative amplification modules were then employed to release the corresponding targets for the next cycle through catalytic hairpin assembly (CHA).

The stepwise assembly of DOF scaffolds was verified through agarose gel electrophoresis (Figure S1, Supporting Information). The migration pattern exhibited a progressive decrease from lane 1 to lane 8 as the DNA strands were introduced, demonstrating the efficient formation of the atom‐like cores of PANs. The formation of PAN reporters was further validated by agarose gel electrophoresis (Figure 1c), which revealed a distinct decrease in electro‐phoretic mobility (lane 3 vs lane 4) upon the addition of the amplification modules (Act‐c and Act‐m), indicating their successful integration onto the DNA frameworks. The atomic force microscopic (AFM) imaging confirmed the octahedral morphology of PAN reporters (Figure 1d). The size of PANs was measured to be ≈12.8 nm, which was consistent with its theoretical length. The cross‐sectional profile of the PANs in Figure 1d along the red line displayed a height of approximately 1.8 nm under the AFM tip (Figure 1e). The size distribution analysis using dynamic light scattering (DLS) in the sample indicated an average hydrated ionic diameter of (23.59 ± 5.72) nm (Figure 1f), exceeding the actual size of PANs due to the formation of hydrogen bond between the DNA hydroxyl groups and H_2_O.^[^ ^14^ ^]^ These results provide further confirmation of the successful formation of the PAN reporters.

### 2.2 PAN Reporters for ncRNA Detection In Vitro

**Figure 2:**
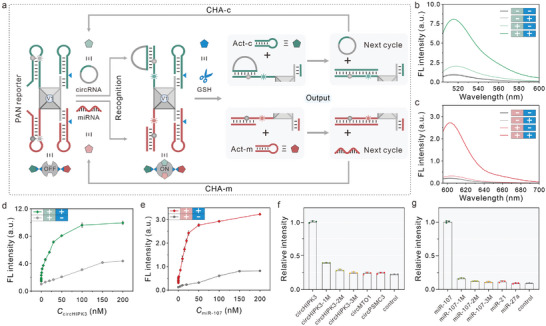
Detection of ncRNAs using PAN reporter. a) Schematic working principle of the CHA strategy for conditionally controlled signal amplification. b) Fluorescent response of PANs to circHIPK‐3 (50 nm) in the absence or presence of 5 mm GSH. c) Fluorescent response of PANs to miR‐107 (50 nm) in the absence or presence of 5 mm GSH. Correlation between fluorescence intensity and the concentration of circHIPK3 d) and miR‐107 e) in the presence or absence of GSH (5 mm), respectively. Specificity of PAN reporters (100 nm) to different circHIPK3 analogs (50 nm) f) and miR‐107 analogs (50 nm) g) with GSH (5 mm). The error bar represents the standard deviation from three independent assays.

We chose circHIPK3 and miR‐107 as targets molecules to assess the viability of PAN reporters. Taking circHIPK3 as an example, we used a short oligo as circHIPK3 mimic to challenge the PAN reporters. Upon the addition of circHIPK3 mimic, the loop area of c‐Probe was hybridized with the target and open the hairpin structure based on strand displacement. As a result, the PAN reporters transition from the “OFF” to the “ON” state, resulting in a detectable change in the fluorescent signal (**Figure** **2**a). Subsequently, Act‐c could be disassembled from PAN reporters in the presence of GSH, thereby initiating a cascade reaction between c‐Probe and Act‐c to generate a robust fluorescence signal of FAM. Consistently, based on strand displacement reactions, the miR‐107 mimic strands can open the corresponding recognition hairpins (m‐Probe), exposing a previously unidentified single‐stranded region that can react with Act‐m and be converted into output signals. The efficacy of recognition modules and amplification modules was verified by the assembly of the DNA duplex. The DNA duplex, c‐Probe/Act‐c and m‐Probe/Act‐m, was only observed when circHIPK3 and miR‐107 were introduced, respectively. The formation of DNA duplex (c‐Probe/Act‐c and m‐Probe/Act‐m) was proven by native polyacrylamide gel electrophoresis (PAGE) analysis (Figure S2, Supporting Information) and fluorescence spectra analysis (Figure S3, Supporting Information), which provides a reference for the subsequent experiments. Furthermore, to verify the validity of PAN reporters, we characterized the responsiveness of the PAN reporters by monitoring changes fluorescence signal intensity. In the absence of circHIPK3 and GSH, only a negligible fluorescence signal of FAM was detected (Figure 2b). However, upon the addition of circHIPK3, a slight increase of fluorescence signal was observed, indicating the locked sensing activity of the amplification modules in PANs due to the presence of a disulfide‐spaced region. Notably, when both circHIPK3 and GSH were present simultaneously, a significant enhancement in the fluorescence intensity of FAM was observed. Similarly, an amplified fluorescence signal of ROX was observed when both miR‐107 and GSH were introduced concurrently (Figure 2c). These observations indicated that the PAN reporters exhibited a notable response to dual targets in the presence of GSH, demonstrating the excellent GSH‐responsive behavior of the disulfide bond. Then, we investigated the effect of GSH concentration on the detection of the PAN reporters in vitro. According to the results, the endogenous GSH at concentration levels in the range of 0.5–10 mm was sufficient to activate the target‐catalyzed CHA reaction (Figure S4, Supporting Information).^[^ ^15^ ^]^ The optimal signal‐to‐noise ratio (S/N) was achieved when the concentration for GSH was 5 mm, which was selected as the optimal reaction concentration of GSH in vitro experiments.

The PAN reporters can trigger a target‐specific CHA reaction in the presence of GSH, leading to a “one‐to‐more” signal amplification. To verify the activation mechanism and the detection performance of the PAN reporters, we investigated the fluorescence response of PANs to different levels of ncRNAs under both GSH‐present and GSH‐absent conditions. Analysis of the fluorescence spectra revealed a gradual increase in the fluorescence intensity of the PANs as the concentration of circHIPK3 and miR‐107 increased from 0 to 200 nm (Figures S5 and S6, Supporting Information). In the presence of GSH, the PAN reporters exhibited higher fluorescence signal intensity due to the initiation of corresponding CHA reactions by circHIPK3 and miR‐107 (Figure 2d,e). As a control, minimal changes in fluorescence signal were observed for PAN reporters in the absence of GSH. The PAN reporters in the presence of GSH demonstrated enhanced sensitivity with a linear range from 0.1 pm to 1 nm (Figure S7, Supporting Information). The limit of detection (LOD) for circHIPK3 and miR‐107 concentrations was estimated to be 234 and 13 fM, respectively. Meanwhile, the PAN reporters without GSH responded to ncRNAs (circHIPK3 and miR‐107) in a broad linear range was 0–150 nm, with the LOD at 7.7 and 0.2 nm, respectively. By contrast, the LOD for “one‐to‐more” signal amplification was four orders of magnitude lower than the condition without signal amplification (Figure S8, Supporting Information). Furthermore, to investigate the selectivity of PAN reporters, we assessed their response to one‐, two‐, or three‐base mismatched sequences, as well as other circ‐HIPK3 analogs (Figure 2f) or miR‐107 analogs (Figure 2g). The results showed that the base mismatched sequence and the tested control circRNAs or miRNAs exhibited relatively low fluorescence signals compared to the target circHIPK3 and miR‐107. These results displayed excellent selectivity of the PAN reporters and demonstrated their potential application in complex cellular environments.

### 2.3 Intracellular Imaging of ncRNAs by PANs‐Based Molecular Classifiers

**Figure 3:**
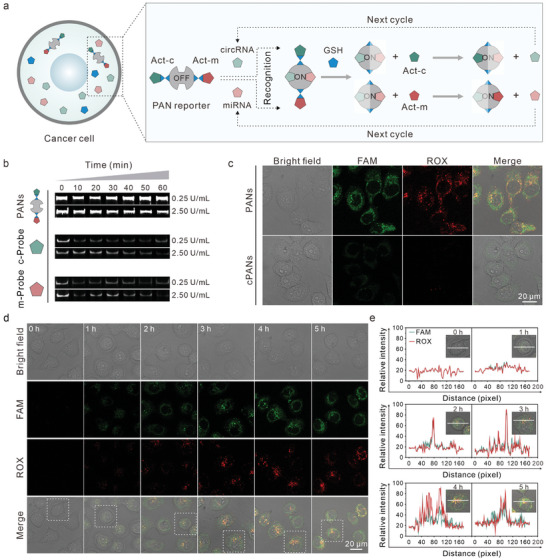
Intracellular imaging of circHIPK3 and miR‐107 using the PANs‐based classifiers. a) Schematic of the PAN reporters simultaneously imaging circHIPK3 and miR‐107 in living cells. b) Electrophoresis analysis for the degradation of PANs and recognition modules (c‐Probe and m‐Probe) treated with low (0.25 U mL^−1^) and high (2.5 U mL^−1^) concentrations of DNase I. c) Confocal microscopic images of SGC‐7901 cells using PANs with (top) and without (bottom) recognition modules, respectively. d) Confocal microscopic images of the PANs dynamically operated in living SGC‐7901 cells within 1–5 h. e) Fluorescence profiling analysis of single cell in the inset figures.

The exploitation of PANs‐based molecular classifiers for intracellular imaging allows for the determination of relative expression information for various ncRNAs in live cells (**Figure** **3**a). Prior to conducting cellular experiments, we evaluated the biostability and cytotoxicity of PAN reporters. The PANs were treated with DNase I with low (0.25 U mL^−1^) and high concentration (2.5 U mL^−1^) for varying time periods. As a comparison, single strands of c‐Probe and m‐Probe were also incubated under identical conditions. After 1 h of treatment, the PANs exhibited no noticeable degradation (Figure 3b), whereas the single strands experienced significant degradation under the same conditions. The grayscale statistical results from ImageJ software showed that over 90% of the PANs maintained structural integrity after being treated with a low concentration of DNase I (0.25 U mL^−1^) for 1 h. In contrast, only ≈25% of the single strands (c‐Probe and m‐Probe) were able to do so (Figure S9a, Supporting Information). Similarly, over 70% of the PANs were able to maintain structural integrity when treated with a high concentration of DNase I (2.50 U mL^−1^) for 1 h, while less than 10% of the single strands (c‐Probe and m‐Probe) could (Figure S9b, Supporting Information). These outcomes clearly demonstrated that the octa hedral scaffold, serving as protective framework core in PANs, successfully shielded the DNA functional modules from nuclease attack.^[^ ^16^ ^]^ Moreover, it was observed that the FAM and ROX fluorescence intensities of the PAN reporters remained virtually unchanged upon incubation with FBS (20% v/v) for 0 to 10 h, indicating that the PAN reporters possess excellent biostability under physiological conditions (Figures S10 and S11, Supporting Information). To assess the cytotoxicity of PAN reporters, an MTT assay was employed. After 48 h of incubation, the cells viability remained above 85% (Figure S12, Supporting Information), thereby confirming the favorable biocompatibility of the PAN reporters.

Then, the imaging capability of PANs‐based molecular classifiers for ncRNAs was validated in living cells. Confocal laser scanning microscopic (CLSM) images in Figure 3c clearly revealed strong fluorescence signals of FAM and ROX in SGC‐7901 cells following incubation with PANs. As control, we constructed cPANs with the same backbone with PAN but substituting recognition modules with a random sequence. Under identical experimental conditions, the cPANs were incubated with SGC‐7901 cells, resulting in negligible fluorescence signals. This observation strongly indicates that the intracellular fluorescence signals generated by PANs are specifically derived from the recognition of circHIPK3 and miR‐107, rather than nonspecific response and degradation‐induced false positives. Moreover, we evaluated the cellular uptake of PANs by treating SGC‐7901 and MKN‐45 cells with FAM‐labeled cPANs (without Dabcyl labeling on Random c‐Probe). Cellular uptake of PANs in these distinct cell types was assessed by confocal imaging and analysis using “ImageJ” software. Similar fluorescence intensities were observed in these cells, implying that they possess equivalent capture efficiency for PANs (Figure S13, Supporting Information). These results validated the effective uptake efficiency of PANs in cells, resulting in reliable outputs of the ncRNA detections. To optimize the incubation time, we performed incubation experiments at various time points (Figure 3d). It was evident that the fluorescence intensities within the cells gradually increased with incubation time, reaching a stable level after 4 h, as indicated by the FAM and ROX signals.^[^ ^17^ ^]^ This finding suggests that a 4 h incubation period is ideal for ncRNAs imaging using PANs. In addition, the fluorescence profiling analysis of individual cells and quantitative analysis of relative fluorescence intensity further confirmed 4 h be the optimal incubation time for ncRNAs imaging (Figure 3e; Figure S14, Supporting Information).

### 2.4 Imaging Analysis of Intracellular circHIPK3 and miR‐107 in Various Cell Lines by PANs‐Based Molecular Classifiers

**Figure 4:**
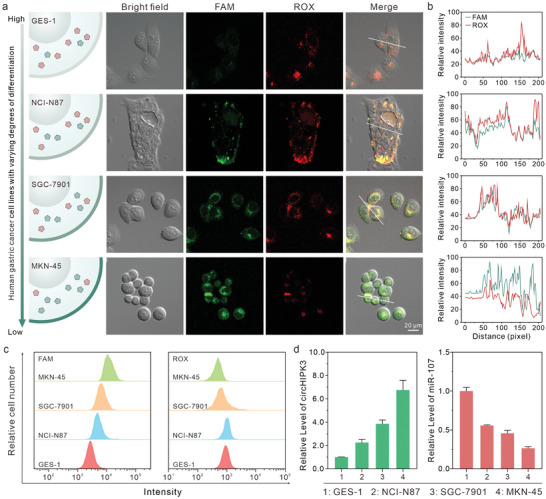
PANs‐based molecular classifiers for imaging multiple ncRNAs in various cell lines. a) Confocal microscopic images of circHIPK3 and miR‐107 in various cell lines, including GES‐1, NCI‐N87, SGC‐7901, and MKN‐45. b) The fluorescence intensity profile along the white line in (a). c) Flow cytometry analysis of the four kinds of cells after incubation with PAN reporters. d) The relative level of circHIPK3 and miR‐107 was determined by qRT‐PCR in the four kinds of cells.

The PANs‐based molecular classifiers were employed to evaluate the expression levels of ncRNAs in a variety of cell types with different degrees of differentiation. We selected human gastric cancer (GC) cell lines, including NCI‐N87 (well differentiated),^[^ ^18^ ^]^ SGC‐7901 (moderately differentiated),^[^ ^19^ ^]^ MKN‐45 (poorly differentiated),^[^ ^20^ ^]^ and normal gastric epidermal cell lines (GES‐1) as our study object. These cell lines represented the two main histopathological types of gastric cancer, namely intestinal type (NCI‐N87) and diffuse type (SGC‐7901, MKN‐45), with the latter exhibiting a higher degree of malignancy.^[^ ^21^ ^]^ In general, there is a negative correlation between tumor malignancy and cellular differentiation. Lower degrees of differentiation are associated with higher malignancy and lower survival rates.^[^ ^22^ ^]^ The CLSM imaging revealed a gradual increase in fluorescence intensity of FAM in GES‐1, NCI‐N87, SGC‐7901, and MKN‐45 cells, indicating a higher concentration of circHIPK3 as cell malignancy increased (**Figure** **4**a,b). Conversely, the fluorescence intensity of ROX decreased in NCI‐N87, SGC‐7901, and MKN‐45 cells, suggesting a lower concentration of miR‐107 as cell malignancy increased. These phenomena implied an association between dys‐regulation of circRNA and miRNA and the degree of cell malignancy and differentiation.^[^ ^23^ ^]^ In addition, the statistics of the relative fluorescence intensity in Figure S15 (Supporting Information) shows that the fluorescence intensities of FAM in MKN‐45 cells were approximately four‐fold higher than GES‐1, while the fluorescence intensities of ROX were only about half of those in GES‐1. These results further validated the findings obtained from the confocal fluorescence imaging.

It is to be noted that the fluorescence intensity of ROX was slightly higher in NCI‐N87 than in GES‐1 (Figure S15, Supporting Information), suggesting that relying solely on the PANs‐based imaging strategy to distinguish the degree of cell differentiation may lead to exceptional cases. This exception can be attributed to several factors. Firstly, normal cells typically display low expression of surface nucleolin, leading to reduced targeting efficiency of PAN reporters toward GES‐1 cells under the same experimental conditions as other GC cells. Secondly, cancer cells show significantly higher intracellular GSH concentrations compared to their corresponding normal cells.^[^ ^24^ ^]^ To verify this, we measured the GSH levels in the cell specimens using a GSH content determination kit based on absorbance at 412 nm and plotted a standard curve (Figure S16, Supporting Information). The GSH levels in NCI‐N87, SGC‐7901, and MKN‐45 cells were determined to be 17.0971, 19.3004, and 19.5596 µg mL^−1^, respectively. In contrast, the GSH level in GES‐1 cells was 9.7095 µg mL^−1^ (Table S2, Supporting Information). These findings suggested that the concentration of GSH in cancer cells was around two fold higher than GES‐1 cells. This characteristic may result in lower levels of GSH in GES‐1 cells, which are necessary to initiate the CHA reactions, thereby influencing the imaging results. The flow cytometry analysis corroborated the finding of the confocal fluorescence imaging (Figure 4c). We performed qRT‐PCR to evaluate the levels of circHIPK3 and miR‐107 in the above cell lines (Figure 4d). This analysis provided a more accurate insight that the expression of circHIPK3 was significantly upregulated and the expression of miR‐107 was significantly downregulated in GC cells. These results further confirmed that the differences in fluorescence observed with the PANs‐based imaging strategy can be attributed to variations in ncRNA content. Moreover, it suggests that the precision of cell classification may be improved by employing multivariate analysis.

### 2.5 Cancer Cell Identification with PANs‐Based Molecular Classifiers

**Figure 5:**
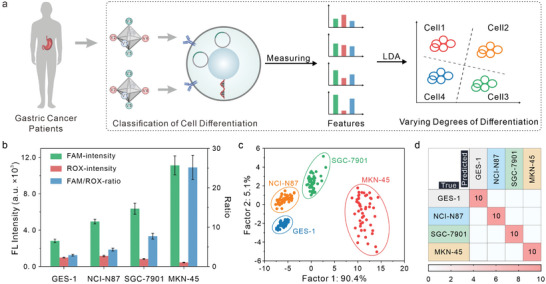
Cancer cell classification with the PANs‐based molecular classifiers. a) Workflow for cell classification using the PAN reporters. b) Fluorescence response patterns of PANs in the four types of cells (10^5^ cell/mL). c) Canonical score plot for the first two factors of fluorescence response obtained from PAN reporters against cells (10^5^ cell mL^−1^), showing the 95% confidence ellipses for individual types of cells. The canonical scores were calculated by linear discriminant analysis (LDA) for the classification of four kinds of cells. d) Detection and classification of unknown cells.

Due to variations in the expression levels of ncRNAs within cells, the interactions between PAN reporters and ncRNAs produce diverse fluorescence responses. To accurately classify different cell lines, we employed a flow cytometer to monitor the fluorescence signal generated by the interactions between PANs‐based molecular classifiers and target ncRNAs in cells, leading to the generation of distinct fingerprints corresponding to each cell type (**Figure** **5**a). Figure 5b illustrates the differential fluoresce responses and FAM/ROX ratio for each cell type, which arise from the recognition and signal amplification of ncRNAs via PAN reporters. The fluorescence responses were measured 50 times for each cell type. The confusion matrix in Tables S3–S5 (Supporting Information) present the results of single‐feature analysis, including FAM‐intensity, ROX‐intensity and FAM/ROX‐ratio, for cell identification. The achieved accuracies were 97.0%, 92.0% and 99.5%, respectively (Figure S17, Supporting Information). Notably, the MKN‐45 and SGC‐7901 were primarily misclassified as other gastric cancer cells and the same was generally true for GES‐1. In comparison, we utilized LDA analysis and visualized the first two most significant factors (Figure 5c), where each point represents the response pattern of the PAN reporters toward a single cell sample. The multidimensional features analysis for cell identification was shown in the confusion matrix in Table S6 (Supporting Information), with an average accuracy of 100%. This improvement suggests that increasing the data dimensionality can enhance the classification power of the system. Furthermore, we conducted a blind experiment to identify another 40 unknown cell samples. The FAM‐intensity analysis and the ROX‐intensity analysis provided accuracies of 95% (38 out of 40 samples, Table S7, Supporting Information) and 87.5% (35 out of 40 samples, Table S7, Supporting Information), respectively. The FAM/ROX‐ratio analysis and the multidimensional features analysis achieved 100% accuracy (Figure 5d; Table S7, Supporting Information), validating the reliability of the biosensor. Thus, our proposed PANs‐based pattern recognition system can function as an intelligent “molecular classifier” for identifying and classifying a wide range of different cells, holding great promise for cancer cell typing and cancer diagnostics.

## 3 Conclusion 

In summary, we have utilized programmable atom‐like nanoparticles (PANs) to develop a versatile molecular classifier with DNA framework as the architecture cores. The applicability of PANs‐based molecular classifiers was demonstrated in various GC cell lines, enabling ultra‐sensitive imaging and classification of cells. With the integration of AS1411 aptamer, PANs can precisely target cancer cells and enhance the internalization efficiency of the reporters into cancer cells. Our versatile PANs possess the capability to detect and image two types of tumor‐related ncRNAs simultaneously in living cells, incorporating a signal amplification strategy based on GSH‐stimulus responsiveness. Consequently, the PAN reporters exhibit minimal false‐positive signals caused by intrinsic biological interferences. Notably, the intrinsic programmability of DNA provides high design flexibility and sequence specificity, the valence and spacing of functional modules in PAN reporters could be effectively programmed in future works. Moreover, the all‐in‐one DNA‐encoded molecular classifiers can be extended to visually analyze various DNAs, viral RNAs, other ncRNAs, and even proteins and small molecules by sequence design, providing a rapid, accurate, and multiplex method for cancer cell identification. Furthermore, it may achieve gene silencing and synergistic treatments by loading siRNA in PAN reporters.^[^ ^25^ ^]^ By further combining with molecules that have a broader spectral range, such as quantum dots, PANs can expand the detection capabilities for multiplex diagnosis of various ncRNA specifications. This novel approach may offer a new tool to analyze multidimensional molecular biomarkers for biological research in cell typing, potentially aiding in precise early cancer diagnosis and treatments.

## Conflict of Interest

The authors declare no conflict of interest.

## Author Contributions

Y.W. and D.Z. conceived and designed the research project. F.Z. designed and fabricated the PANs. F.Z., X.Y. and L.O. analyzed the results. T.M. and S.D. helped perform the analysis with constructive discussions, F.Z., Y.W., D.Z., and J.C. wrote the paper, and all authors read and approved the manuscript.

## Supporting information

Supporting Information

## Data Availability

The data that support the findings of this study are available from the corresponding author upon reasonable request.

## References

[1] a) G. A. Calin , C. M. Croce , Nat. Rev. Cancer 2006, 6, 857; 17060945 10.1038/nrc1997

[2] a) P. D. Robbins , A. E. Morelli , Nat. Rev. Immunol. 2014, 14, 195; 24566916 10.1038/nri3622 PMC4350779

[3] a) J. Wei , H. Xu , W. Wei , Z. Wang , Q. Zhang , W. De , Y. Shu , Onco Targets Ther 2020, 13, 1613; 32110057 10.2147/OTT.S226300 PMC7041611

[4] a) L. S. Kristensen , M. S. Andersen , L. V. W. Stagsted , K. K. Ebbesen , T. B. Hansen , J. Kjems , Nat. Rev. Genet. 2019, 20, 675; 31395983 10.1038/s41576-019-0158-7

[5] C. Xue , S. X. Zhang , C. H. Ouyang , D. Chang , B. J. Salena , Y. Li , Z. S. Wu , Angew. Chem., Int. Ed. 2018, 57, 9739. 10.1002/anie.201804741 29901854

[6] a) G. Obernosterer , J. Martinez , M. Alenius , Nat. Protoc. 2007, 2, 1508; 17571058 10.1038/nprot.2007.153

[7] a) J. Yan , C. Hu , P. Wang , B. Zhao , X. Ouyang , J. Zhou , R. Liu , D. He , C. Fan , S. Song , Angew. Chem., Int. Ed. 2015, 54, 2431; 10.1002/anie.201408247 25599663

[8] a) Y. Wang , Y. Bai , L. P. Cao , L. L. Li , L. Zhan , H. Zuo , C. M. Li , C. Z. Huang , Biosens. Bioelectron. 2022, 197, 113783; 34775254 10.1016/j.bios.2021.113783

[9] a) F. Yin , H. Zhao , S. Lu , J. Shen , M. Li , X. Mao , F. Li , J. Shi , J. Li , B. Dong , W. Xue , X. Zuo , X. Yang , C. Fan , Nat. Nanotechnol. 2023, 18, 677; 36973399 10.1038/s41565-023-01348-9

[10] D. Samanta , W. Zhou , S. B. Ebrahimi , S. H. Petrosko , C. A. Mirkin , Adv. Mater. 2022, 34, 2107875. 10.1002/adma.202107875 34870875

[11] a) J. Li , J. Dai , S. Jiang , M. Xie , T. Zhai , L. Guo , S. Cao , S. Xing , Z. Qu , Y. Zhao , F. Wang , Y. Yang , L. Liu , X. Zuo , L. Wang , H. Yan , C. Fan , Nat. Commun. 2020, 11, 2185; 32366822 10.1038/s41467-020-16112-z PMC7198603

[12] a) Z. Jiang , N. D. B. Le , A. Gupta , V. M. Rotello , Chem. Soc. Rev. 2015, 44, 4264; 25853985 10.1039/c4cs00387j PMC4478158

[13] a) E. M. Reyes‐Reyes , Y. Teng , P. J. Bates , Cancer Res. 2010, 70, 8617; 20861190 10.1158/0008-5472.CAN-10-0920 PMC2970734

[14] P. Zhang , J. Jiang , R. Yuan , Y. Zhuo , Y. Chai , J. Am. Chem. Soc. 2018, 140, 9361. 30008212 10.1021/jacs.8b04648

[15] a) M. H. Lee , Z. Yang , C. W. Lim , Y. H. Lee , S. Dongbang , C. Kang , J. S. Kim , Chem. Rev. 2013, 113, 5071; 23577659 10.1021/cr300358b

[16] a) D. Wang , R. Peng , Y. Peng , Z. Deng , F. Xu , Y. Su , P. Wang , L. Li , X.‐Q. Wang , Y. Ke , W. Tan , ACS Nano 2020, 14, 17365; 36350012 10.1021/acsnano.0c07495

[17] a) H. Wang , P. Peng , Q. Wang , Y. Du , Z. Tian , T. Li , Angew. Chem., Int. Ed. 2020, 59, 6099; 10.1002/anie.201916432 31981393

[18] H. O. Duarte , M. Balmana , S. Mereiter , H. Osorio , J. Gomes , C. A. Reis , Int. J. Mol. Sci. 2017, 18, 2262. 29143776 10.3390/ijms18112262 PMC5713232

[19] W. J. Jiang , L. Y. Zhou , S. R. Lin , Y. Li , S. Y. Xiao , J. Liu , Z. P. Li , Y. Cui , J. Z. Zhang , Int. J. Clin. Exp. Pathol. 2018, 11, 869. 31938177 PMC6958048

[20] Saikawa , Int J Oncol 2009, 34, 1201. 19360333

[21] a) Y. Cui , S. B. Li , X. C. Peng , J. Wu , G. H. Fu , Dig Dis Sci 2015, 60, 3631; 26173505 10.1007/s10620-015-3793-7

[22] a) Z. Yang , J. Xu , L. Li , R. Li , Y. Wang , Y. Tian , W. Guo , Z. Wang , F. Tan , J. Ying , Y. Jiao , S. Gao , J. Wang , Y. Gao , J. He , Nat. Commun. 2020, 11, 4878; 32985499 10.1038/s41467-020-18702-3 PMC7522294

[23] a) M. Su , Y. H. Xiao , J. L. Ma , Y. Y. Tang , B. Tian , Y. Q. Zhang , X. Li , Z. N. Wu , D. S. Yang , Y. Zhou , H. Wang , Q. J. Liao , W. X. Wang , Mol Cancer 2019, 18, 1; 30999909 10.1186/s12943-019-1002-6 PMC6471953

[24] a) X. Liu , Y. Li , K. Wang , Y. Chen , M. Shi , X. Zhang , W. Pan , N. Li , B. Tang , Nano Lett. 2021, 21, 7862; 34494442 10.1021/acs.nanolett.1c03089

[25] a) X. Wang , X. Shen , J. Li , X. Ge , J. Ouyang , N. Na , Anal. Chem. 2022, 94, 16803; 36342409 10.1021/acs.analchem.2c03726

