# Supplementary material for: DNA Framework‐Based Programmable Atom‐Like Nanoparticles for Non‐Coding RNA Recognition and Differentiation of Cancer Cells
Source: Adv Sci (Weinh). 2024 Apr 3;11(23):2400492. doi: 10.1002/advs.202400492 (PMC11187905; doi:10.1002/advs.202400492)
Supplement: Supplementary file 1 — Supporting Information [file ADVS-11-2400492-s001.pdf]

ADVANCED SCIENCE
Open Access

Supporting Information

for *Adv. Sci.*, DOI 10.1002/advs.202400492

DNA Framework-Based Programmable Atom-Like Nanoparticles for Non-Coding RNA Recognition and Differentiation of Cancer Cells

Fulin Zhu, Xinyu Yang, Lilin Ouyang, Tiantian Man, Jie Chao, Shengyuan Deng, Dan Zhu* and Ying Wan**

Supporting Information

DNA Framework-Based Programmable Atom-like Nanoparticles for Non-Coding RNA Recognition and Differentiation of Cancer Cells

Fulin Zhu,[1] Xinyu Yang,[1] Lilin Ouyang,[2] Tiantian Man,[1] Jie Chao,[2] Shengyuan Deng,[3,*] Dan Zhu,[2,*] and Ying Wan,[1,*]

1 Dr. F. Zhu, X. Yang, Prof. T. Man, Prof. Y. Wan

School of Mechanical Engineering, Nanjing University of Science and Technology, 200 Xiaolingwei Street, Nanjing, 210094, China

E-mail: wanying@njust.edu.cn

2 Dr. L. Ouyang, Prof. J. Chao, Prof. D. Zhu

State Key Laboratory of Organic Electronics and Information Displays & Jiangsu Key Laboratory for Biosensors, Institute of Advanced Materials (IAM), Jiangsu National Synergetic Innovation Center for Advanced Materials (SICAM), Nanjing University of Posts and Telecommunications, 9 Wenyuan Road, Nanjing, 210023, China

E-mail: iamdzhu@njupt.edu.cn

3 Prof. S. Deng

School of Environmental and Biological Engineering, Nanjing University of Science and Technology, 200 Xiaolingwei Street, Nanjing, 210094, China

E-mail: sydeng@njust.edu.cn

CONTENT

1. Supplementary Materials and Reagents
2. Supplementary Experimental Methods
3. Supplementary Figures
4. Supplementary Table

1. Supplementary Materials and Reagents

All DNA oligonucleotides were synthesized and purified by Sangon Biotech Co., Ltd. (Shanghai, China), and their sequences are presented in Table S1. Glutathione (GSH) and the reduced GSH content assay kit (Micromethod) were purchased from Sangon Biotech Co., Ltd. (Shanghai, China). Normal human gastric epithelial cells (GES-1), the poorly differentiated gastric cancer cells (MKN-45) and the high differentiated gastric cancer cells (NCI-N87) were obtained from KeyGEN Biotech Co., Ltd. (Nanjing, China). The moderately differentiated gastric cells (SGC-7901) were obtained from Beyotime Biotechnology (Nantong, China). Fetal bovine serum (FBS) was provided by Gibco though Life Technology (New York, USA). The DNase I, 3-(4,5-Dimethylthiazol-2-yl)-2,5-diphenyltetrazolium bromide (MTT) and RPMI-1640 medium (10% penicillin-streptomycin) were purchased from KeyGEN Biotech Co., Ltd. (Nanjing, China). All other reagents were of analytical grade and used as received without further purification. Ultrapure water ($\geq$18.2MΩ cm) was used throughout the study.

2. Supplementary Experimental Methods

Self-assembly of the PANs. The DNA concentration was quantified by the measurement of the absorbance at 260 nm (UV-3600, Shimadzu, Japan). To prepare the DNA octahedron, eight DNA strands (S1~S6, c-Probe, and m-Probe, Table S1) were mixed in 1×Tris-buffer (20 mM Tris, 250 mM NaCl, 100 mM KCl, 10 mM Mg^{2+}, pH 8.0). The mixture was heated at 95 ºC for 10 min, then cooled down to 4 ºC slowly at a speed of -1 ºC per 3 min on a PCR instrument. Simultaneously, the DNA strands Act-c and Act-m were annealed at 95 ºC for 10 min and then cooled to room temperature gradually for forming the hairpin nanostructure. The PAN reporters were formed by incubating Act-c and Act-m both with DNA octahedron at 25 ºC for 1 h and stored at 4 ºC for further use. The final concentration of each oligonucleotide was 1 μM.

Electrophoresis analysis. Nondenaturing polyacrylamide gel electrophoresis (PAGE) was performed for the analysis of the CHA products. The samples (10 μL) and 6× loading buffer (2 μL) were loaded into this 12% nondenaturing polyacrylamide gel. Electrophoresis was performed in 1× TBE buffer at 100 V for 90 min. Following, the gels were stained with GelRed and imaged on the gel imaging system (Tanon Science & Technology Co., Ltd, China).

Agarose gel electrophoresis (AGE) was performed for the analysis of the PAN formation. A volume of 5 μL of the samples was mixed with 1 μL of 6× loading buffer and added to a 2% agarose gel with GelRed. The electrophoresis experiments were conducted in 1× TAE buffer at 100 V for 50 min. After that, the gel was imaged using the gel imaging system (Tanon Science & Technology Co., Ltd, China).

Fluorescence measurements. Fluorescence measurement was performed with a fluorescence S-3 spectrophotometer (RF-5301PC, Shimadzu, Japan) for monitoring the PAN reporters signal amplification process of circHIPK3 and miR-107 in the presence of GSH (5 mM). The excitation wavelengths were set at 490 nm for FAM and 580 nm for ROX, respectively. The PAN reporters (final concentration of 100 nM) were mixed with targets ncRNA (circHIPK3 and miR-107) in 1× Tris-buffer. After incubation for 3 h, the changed fluorescence was recorded. Finally, the fluorescence spectra were normalized (a.u.). All experiments were repeated at least 3 times.

Stability assay. To evaluate the nuclease stability of the PAN reporters, low (0.25 U/mL) and high (2.5 U/mL) concentration of DNase I was respectively added to the sample

solution containing 250 nM PAN reporters and incubated with different time (0, 10, 20, 30, 40, 50, 60 min) at 37 °C. As control group, same process was applied to the reporter units (c-Probe and m-Probe). The degradative mixtures were characterized by PAGE imaging. To further evaluate the serum stability of the PAN reporters, the 100 nM PAN reporters and 100 nM recognition elements (c-Probe and m-Probe) were added into 10% (v/v) FBS, respectively. The time-dependent fluorescence spectrum was monitored for 10 h.

Cell culture. Human gastric cancer cell lines (NCI-N87, SGC-7901, MKN-45) and normal gastric epidermal cell lines (GES-1) were cultured in RPMI-1640 medium supplemented with 10% FBS. All cells were maintained at 37 °C in humidified environment containing 5% CO_2.

Evaluation of cellular cytotoxicity of PAN reporters. The cytotoxicity of the PAN reporters was determined by a standard MTT assay. The SGC-7901 cells (1×10^4 cells per well) were seeded in 96-well culture plates at 37 °C for 18 h. Then the SGC-7901 cells were treated with PAN reporters (100 nM and 500 nM) and incubated for different times (3 h, 6 h, 12 h, 24 h, 36 h and 48 h). Subsequently, the cell culture medium was replaced by MTT solutions (5 mg/mL) and incubated at 37 °C for 4 h. Finally, 100 μL of dimethyl sulfoxide was added after removing the MTT solutions, and the absorbance was measured at 490 nm with a microplate reader (EL × 808, BioTek) in order to calculate cell viability.

Confocal microscopy assay. The human gastric cancer cell lines (NCI-N87, SGC-7901, MKN-45) and normal gastric epidermal cell lines (GES-1) were respectively seeded into 15 mm confocal dishes for 24 h at 37 °C with 5% CO_2. Then the medium was removed and washed three times with 1× D-PBS. The culture medium containing PAN reporters (100 nM) was added in the dishes and incubated at 37 °C for 4 h. After that, the cells were washed three times with 1× D-PBS and observed under a laser-scanning confocal microscopy system (A1 + SIM S, Nikon, Japan). The fluorescent imaging of FAM (circHIPK3) in cells was performed in the green channel with 488 nm excitation, and that of ROX (miR-107) was performed in the red channel with 559 nm excitation. The fluorescence intensity of each sample was analyzed with ImageJ software.

Flow cytometry analysis. In the cancer cell identification study, 100 nM PAN reporters were incubated with different cells (1×10^5 cells/mL) for 4 h at 37 °C with 5% CO_2.

After that, the cells were detached from culture dishes using trypsin solution. Then, the mixture was centrifuged under 800 rpm for 5 min and resuspended in 1× D-PBS for three times. Finally, the fluorescence intensity of the cell population was then determined using flow cytometry (FC500, Beckman Coulter, United States). The FAM-intensity, ROX-intensity and FAM/ROX-ratio were recorded, respectively. Thus, the PAN reporters against four types of cells were tested fifty times in the same condition, giving a 3×4×50 training data matrix. The raw data matrix was processed using classical linear discriminant analysis (LDA).

NcRNAs Quantitation by qRT-PCR. First, total RNAs were extracted from human gastric cancer cell lines (NCI-N87, SGC-7901, MKN-45) and normal gastric epidermal cell lines (GES-1) with RNA trizol reagents, respectively. Subsequently, the cDNA samples were prepared through reverse transcription reaction utilizing the AMV First Strand cDNA Synthesis Kit (BBI, Toronto, Canada). The expression levels of circHIPK3 and miRNA-107 were analyzed using SG Fast qPCR Master Mix (2×; BBI), following the protocol specified in the LightCycler480 software settings (Roche Ltd., Basel, Switzer-land). The relative expression level of circHIPK3 or miRNA-107 was determined using the 2-ΔΔCt method. All RT-PCR reactions were repeated at least three times.

3. Supplementary Figures

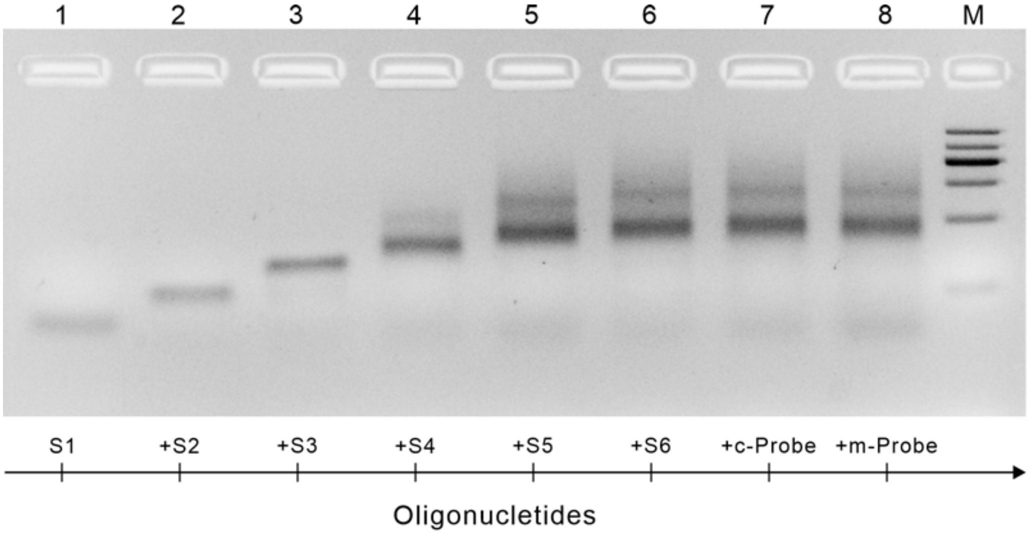

Figure S1. AGE analysis of stepwise assembly DOFs.

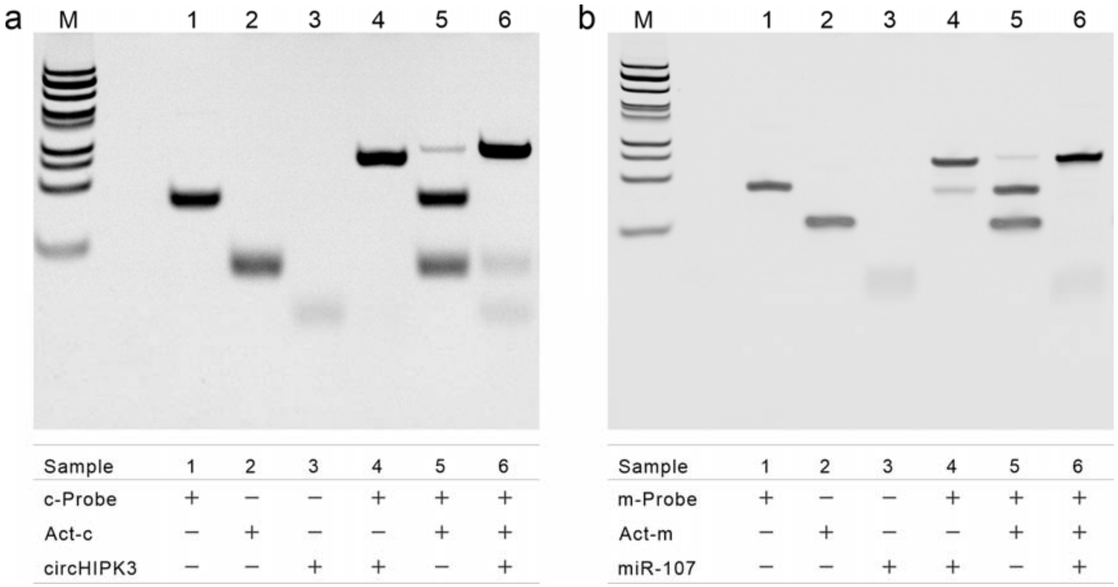

Figure S2. (a) Characterization of CHA-c by PAGE. Lane1-6 represent c-Probe, Act-c, circHIPK3, c-Probe + circHIPK3, c-Probe + Act-c, c-Probe + Act-c + circHIPK3. (b) Characterization of CHA-m by PAGE. Lane1-6 represent m-Probe, Act-m, miR-107, m-Probe + miR-107, m-Probe + Act-m, m-Probe + Act-m+ miR-107.

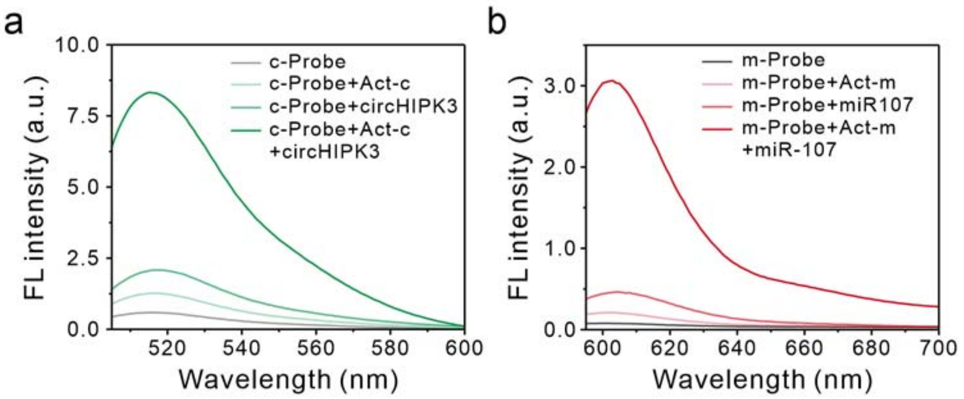

Figure S3. (a) The fluorescence spectra of the circHIPK3-triggered CHA-c reaction. The concentration of circHIPK3 was 50 nM, and the concentration of c-Probe and Act-c were 100 nM. (b) The fluorescence spectra of the miR-107-triggered CHA-m reaction. The concentration of miR-107 was 50 nM, and the concentration of m-Probe and Act-m were 100 nM.

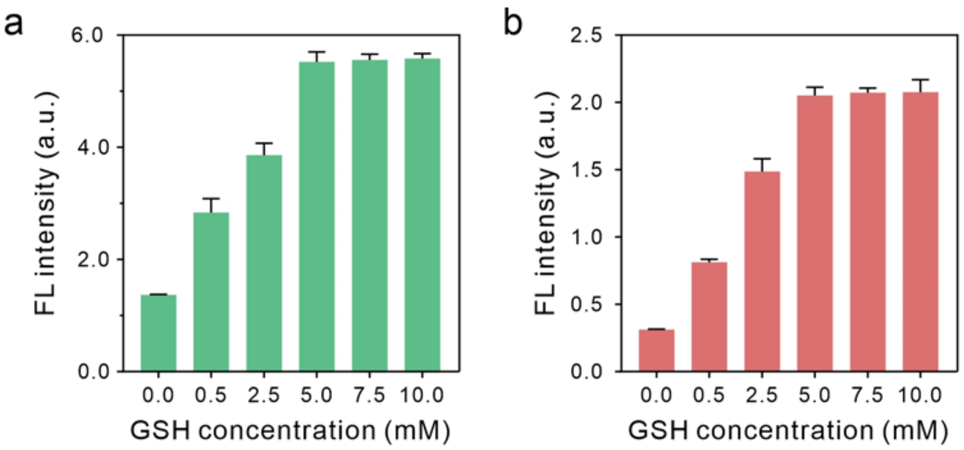

Figure S4. Fluorescence intensity of PAN reporters (100 nM) after being incubated with (a) circHIPK3 (20 nM) and (b) miR-107 (20 nM) in the presence of different concentrations (0 mM, 0.5 mM, 2.5 mM, 5.0 mM, 7.5 mM, 10 mM) of GSH *in vitro*.

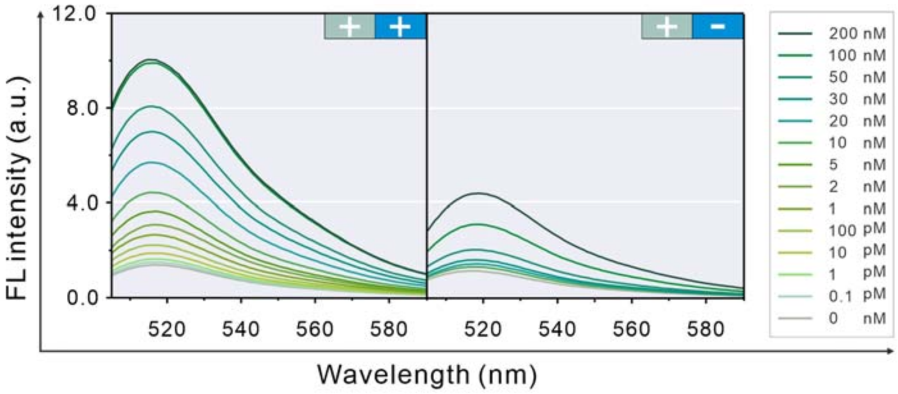

Figure S5. Fluorescence spectra of PAN reporters toward analyzing various concentrations of circHIPK3 in the presence or absence of GSH (5 mM).

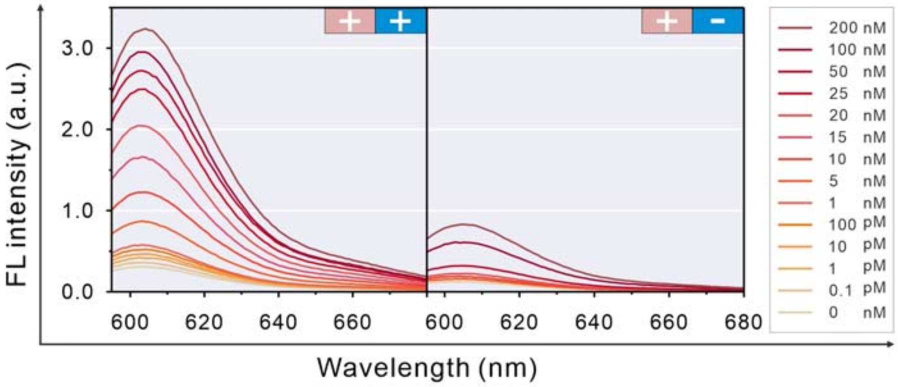

Figure S6. Fluorescence spectra of PAN reporters toward analyzing various concentrations of miR-107 in the presence or absence of GSH (5 mM).

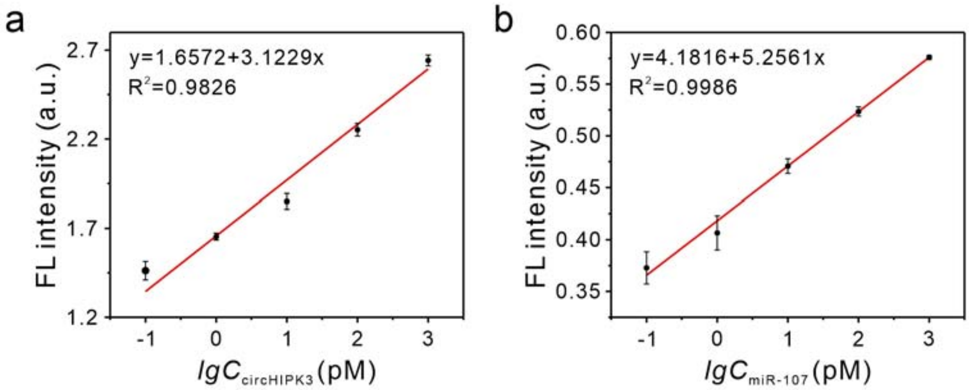

Figure S7. (a) The linear relationship between the maximum fluorescence intensities and circHIPK3 concentrations based on CHA strategy. (b) The linear relationship between the maximum fluorescence intensities and miR-107 concentrations based on CHA strategy.

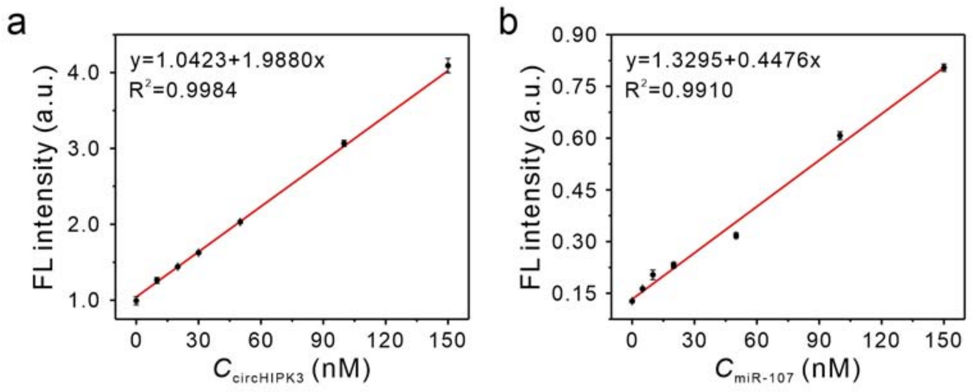

Figure S8. (a) The linear relationship between the maximum fluorescence intensities and circHIPK3 concentrations without CHA strategy. (b) The linear relationship between the maximum fluorescence intensities and miR-107 concentrations without CHA strategy

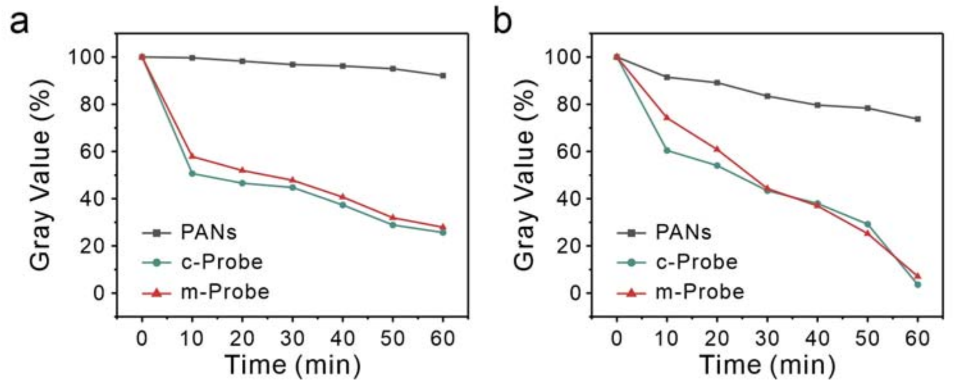

Figure S9. The grayscale statistical results of degradation rate of PANs and recognition modules (c-Probe and m-Probe) treated with low concentration of DNase I (0.25 U/mL) (a) and high concentration of DNase I (2.5 U/mL) (b).

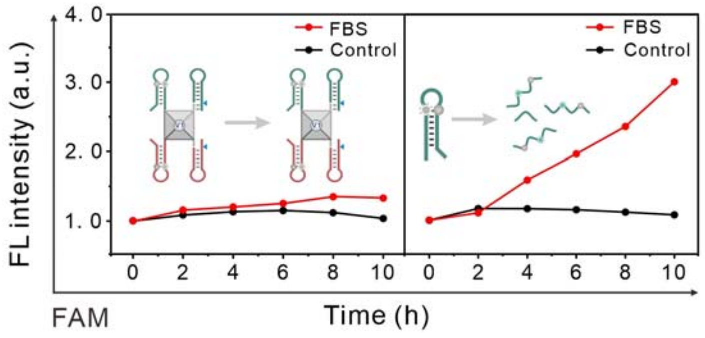

Figure S10. The fluorescence intensities of FAM in 20% FBS solution after incubating with PAN reporters and the nude c-Probes from 0 h to 10 h.

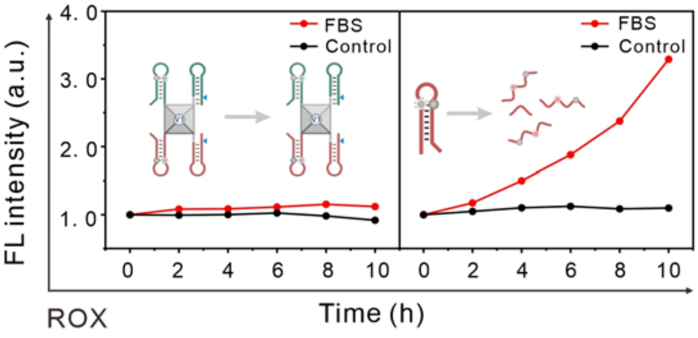

Figure S11. The fluorescence intensities of ROX in 20% FBS solution after incubating with PAN reporters and the nude m-Probes from 0 h to 10 h.

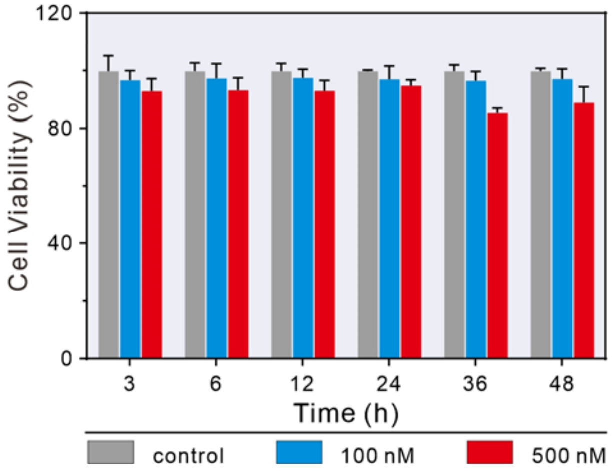

Figure S12. Viability of SGC-7901 cells treated with different concentration of PAN reporters.

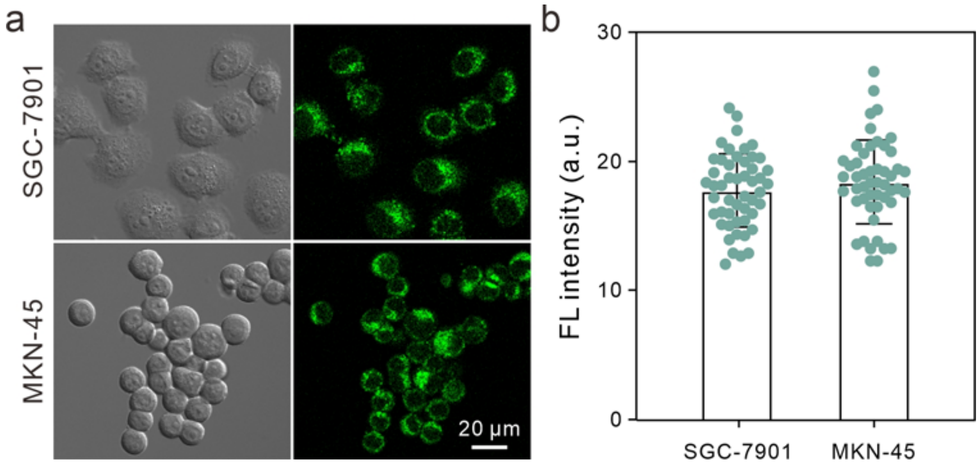

Figure S13. (a) Confocal microscopic images of SGC-7901 and MKN-45 cells after being treated with FAM-labeled cPANs (without Dabcyl-labeled on Random c- Probe). (b) Quantification of the mean fluorescence intensity in (a). Data are means ±SD (n = 50 cells).

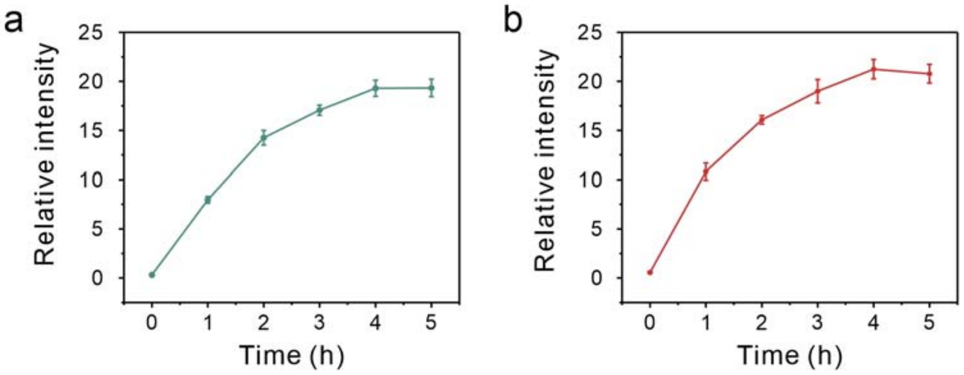

Figure S14. The corresponding relative fluorescence intensity of SGC-7901 cells incubated with 100 nM PAN reporters for FAM (a) and ROX (b) at different times (0, 1, 2, 3, 4, 5 h).

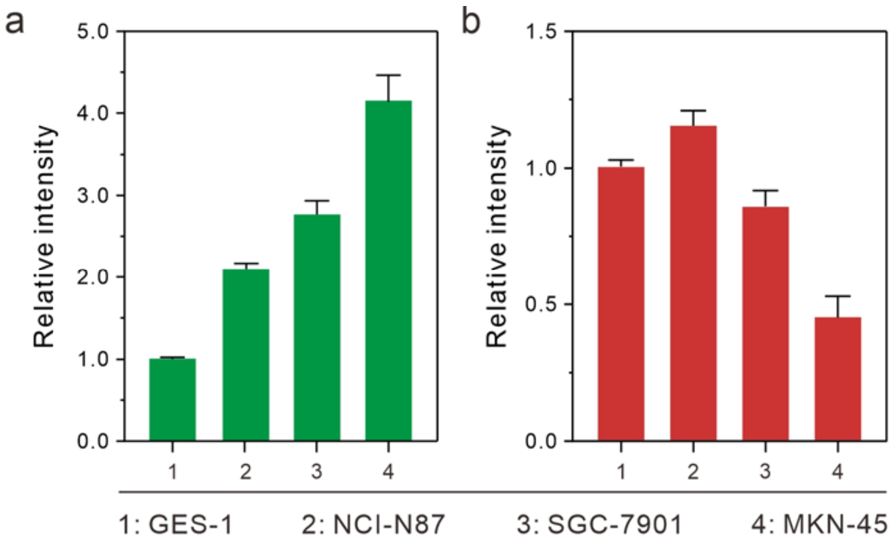

Figure S15. Histogram of the corresponding relative fluorescence intensity of FAM (a) and ROX (b) in Figure 4a.

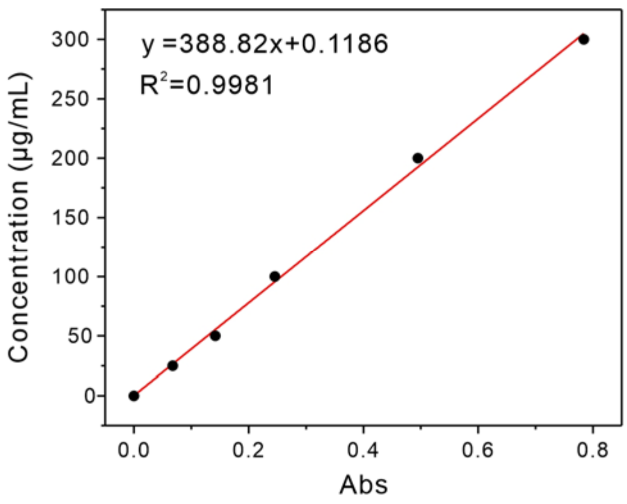

Figure S16. Linear fitting curve of the standard glutathione kit.

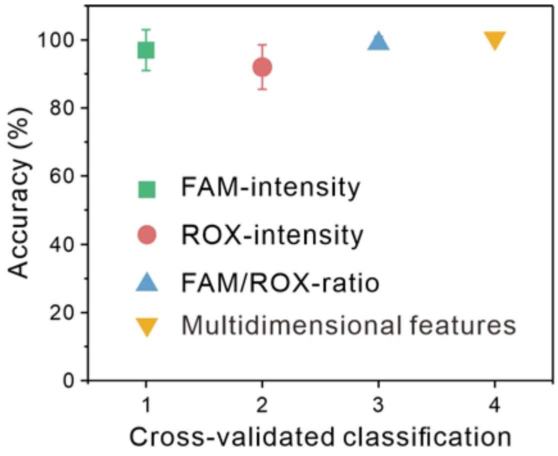

Figure S17. Accuracy of cross-validated classification.

4. Supplementary Tables

Table S1. DNA sequence used in this study

Name	Sequence(5'to3')
**S1**	CGGTCATCGTCGTCGTCGTTGCACGAATACGAATACTATTCGCAAGAATTTAGGATCCTTGTCATTT
**S2**	GGATACATTATACGGTGGTTTGTACGAGCATAGGATTCTTCCTGGTGAAGTATGCCAATTGGATCCT
**S3**	CCACCGTATAATGTATCCTTCGAGCAGCACGAACTGTCTTCATTCGTCGTCGTCGTAGTTCTGTAGCCTCTTATGCGGTT*TCACGAGTAAGGCTATCA*
**S4**	TTGGCATACTTCACCAGGTTGAATCCTATGCTCGTACATTGTCGCAGTTCAGATACGCTTCATACTGAGAGCGTTCCGTT*TGTCTGCGAACGGTACTA*
**S5**	GCGTATCTGAACTGCGACTTCCGCATAAGAGGCTACAGTTGGACCGTAGTTAAATGACTTCGGAACGCTCTCAGTATGTTTTT***GGTGGTGGTGGTTGTGGTGGTGGTGG***
**S6**	CGACGACGACGATGACCGTTCTACGACGACGACGAATGTTGACAGTTCGTGCTGCTCGTTTAGTATTCGTATTCGTGCTTTTT***GGTGGTGGTGGTTGTGGTGGTGGTGG***
**c-Probe**	AAATTCTTGCGTTTTTCAATCTCGGTAC-FAM-AAGATGTGTACC-Dabcyl-AGTACCGAGATTGTAGATATG
**m- Probe**	AACTACGGTCCTTTTTTGTACAGGGCTA-ROX-CATTGAATCCAGAG-BHQ2-GATAGCCCTGTACAATGCTGCT
**Act-c**	AGATGTGTACCACAATCTCGGTACTAGGTACACATCT*TAGTACCG*-S-S-*TTCGCAGACA*
**Act-m**	TTGAATCCAGAGTTGTACAGGGCTATCACTCTGGATTCAA*TGATAGGC*-S-S-*TTACTCGTGA*
**Random c- Probe**	AAATTCTTGCGTTTTTGCAGAGTGGAGGT-FAM-AGCGAGTATGTTT-Dabcyl-ACCTCCACTCTGCTACCATCA
**Random m- Probe**	AACTACGGTCCTTTTTCAGACTACCAAGAT-ROX-ACAATGCAAGGGTT-BHQ2-ATCTTGGTAGTCTGGTCATATA
**Random c- Probe***	AAATTCTTGCGTTTTTGCAGAGTGGAGGT-FAM-AGCGAGTATGTTTACCTCCACTCTGCTACCATCA

**miR-107**	AGCAGCATTGTACAGGGCTATCA
**circHIPK3**	CATATCTACAATCTCGGTACTA
**miR-107-1M**	AGCACCATTGTACAGGGCTATCA
**miR-107-2M**	AGCACCATTGTCCAGGGCTATCA
**miR-107-3M**	AGCACCATTGTCCAGGGATATCA
**miR-21**	TAGCTTATCAGACTGATGTTGA
**miR-27**	TTCACAGTGGCTAAGTTCCGC
**circHIPK3-1M**	CATACCTACAATCTCGGTACTA
**circHIPK3-2M**	CATACCTACAACCTCGGTACTA
**circHIPK3-3M**	CATACCTACAACCTCGGAACTA
**circMTO1**	GTGGGGTTGTTTTGGGTCAGATGTCATGTA
**circPSMC3**	GGGTCCCTGCCCTTTGACAGTG

Table S2. The results of GSH concentration measurement using a GSH content determination kit

Cells (10^6/mL)	Mean Abs (412 nm)	Concentration of GSH (μg/mL）
GES-1	0.0247	9.7095
NCI-N87	0.0437	17.0971
SGC-7901	0.0493	19.3004
MKN-45	0.0500	19.5596

Table S3. Cross-validation of LDA on the data set using FAM intensity.

	I	II	III	IV	Correct (%）
I	50	0	0	0	100
II	0	50	0	0	100
III	0	0	50	0	100
IV	0	0	6	44	88.0
Total	50	50	56	44	97.0

I: GES-1 II: NCI-N87 III: SGC-7901 IV: MKN-45

Table S4. Cross-validation of LDA on the data set using ROX intensity.

	I	II	III	IV	Correct (%）
I	46	4	0	0	92.0
II	0	50	0	0	100
III	8	0	42	0	84.0
IV	4	0	0	46	92.0
Total	58	50	46	46	92.0

I: GES-1 II: NCI-N87 III: SGC-7901 IV: MKN-45

Table S5. Cross-validation of LDA on the data set using FAM/ROX ratio.

	I	II	III	IV	Correct (%）
I	50	0	0	0	100
II	0	50	0	0	100
III	1	0	49	0	98.0
IV	0	0	0	50	100
Total	51	50	49	50	99.5

I: GES-1 II: NCI-N87 III: SGC-7901 IV: MKN-45

Table S6. Cross-validation of LDA on the data set using Dimensionality Reduction Features.

	I	II	III	IV	Correct (%）
I	50	0	0	0	100
II	0	50	0	0	100
III	0	0	50	0	100
IV	0	0	0	50	100
Total	50	50	50	50	100

I: GES-1 II: NCI-N87 III: SGC-7901 IV: MKN-45

Table S7. Detection and identification of unknown cells with PAN reporters

Samples	PANs reporters			Identification	Verification by FAM-intensity	Verification by ROX-intensity	Verification by FAM/ROX-ratio	Verification by multidimensional features
	FAM-intensity	ROX-intensity	FAM/ROX-ratio					
1	2974	861	3.454	GES-1	GES-1	**SGC-7901**	GES-1	GES-1
2	2912	857	3.398	GES-1	GES-1	**SGC-7901**	GES-1	GES-1
3	2758	828	3.331	GES-1	GES-1	**SGC-7901**	GES-1	GES-1
4	2853	917	3.111	GES-1	GES-1	GES-1	GES-1	GES-1
5	2753	992	2.775	GES-1	GES-1	GES-1	GES-1	GES-1
6	2865	1014	2.825	GES-1	GES-1	GES-1	GES-1	GES-1
7	2783	964	2.887	GES-1	GES-1	GES-1	GES-1	GES-1
8	2922	938	3.115	GES-1	GES-1	GES-1	GES-1	GES-1
9	2945	956	3.081	GES-1	GES-1	GES-1	GES-1	GES-1
10	2924	954	3.065	GES-1	GES-1	GES-1	GES-1	GES-1
11	4029	1012	3.981	NCI-N87	NCI-N87	**GES-1**	NCI-N87	NCI-N87
12	5430	1122	4.840	NCI-N87	NCI-N87	NCI-N87	NCI-N87	NCI-N87
13	5200	1197	4.344	NCI-N87	NCI-N87	NCI-N87	NCI-N87	NCI-N87
14	5989	1353	4.426	NCI-N87	**SGC-7901**	NCI-N87	NCI-N87	NCI-N87
15	4824	1065	4.530	NCI-N87	NCI-N87	**GES-1**	NCI-N87	NCI-N87
16	5228	1258	4.156	NCI-N87	NCI-N87	NCI-N87	NCI-N87	NCI-N87
17	4408	1091	4.040	NCI-N87	NCI-N87	NCI-N87	NCI-N87	NCI-N87
18	5604	1219	4.597	NCI-N87	NCI-N87	NCI-N87	NCI-N87	NCI-N87
19	5395	1290	4.182	NCI-N87	NCI-N87	NCI-N87	NCI-N87	NCI-N87
20	5537	1293	4.282	NCI-N87	NCI-N87	NCI-N87	NCI-N87	NCI-N87
21	5554	794	6.995	SGC-7901	**NCI-N87**	SGC-7901	SGC-7901	SGC-7901
22	5692	807	7.053	SGC-7901	SGC-7901	SGC-7901	SGC-7901	SGC-7901
23	6792	890	7.631	SGC-7901	SGC-7901	SGC-7901	SGC-7901	SGC-7901
24	6619	864	7.661	SGC-7901	SGC-7901	SGC-7901	SGC-7901	SGC-7901
25	6130	804	7.624	SGC-7901	SGC-7901	SGC-7901	SGC-7901	SGC-7901

26	6408	833	7.693	SGC-7901	SGC-7901	SGC-7901	SGC-7901	SGC-7901
27	6645	853	7.790	SGC-7901	SGC-7901	SGC-7901	SGC-7901	SGC-7901
28	6494	834	7.787	SGC-7901	SGC-7901	SGC-7901	SGC-7901	SGC-7901
29	6192	850	7.285	SGC-7901	SGC-7901	SGC-7901	SGC-7901	SGC-7901
30	6351	873	7.275	SGC-7901	SGC-7901	SGC-7901	SGC-7901	SGC-7901
31	11150	463	24.082	MKN-45	MKN-45	MKN-45	MKN-45	MKN-45
32	12006	507	23.680	MKN-45	MKN-45	MKN-45	MKN-45	MKN-45
33	11888	478	24.870	MKN-45	MKN-45	MKN-45	MKN-45	MKN-45
34	11136	434	25.659	MKN-45	MKN-45	MKN-45	MKN-45	MKN-45
35	10967	477	22.992	MKN-45	MKN-45	MKN-45	MKN-45	MKN-45
36	12456	467	26.672	MKN-45	MKN-45	MKN-45	MKN-45	MKN-45
37	10165	485	20.959	MKN-45	MKN-45	MKN-45	MKN-45	MKN-45
38	11918	386	30.876	MKN-45	MKN-45	MKN-45	MKN-45	MKN-45
39	10577	479	22.081	MKN-45	MKN-45	MKN-45	MKN-45	MKN-45
40	11733	422	27.803	MKN-45	MKN-45	MKN-45	MKN-45	MKN-45

Identification accuracy: 95.0% (38/40) using FAM-intensity; 87.5% (35/40) using ROX-intensity; 100% (40/40) using FAM/ROX-ratio; 100% (40/40) using multidimensional features. Red bold indicates the incorrectly identified samples.
